# Identification of Whole-Serum Glycobiomarkers for Colorectal Carcinoma Using Reverse-Phase Lectin Microarray

**DOI:** 10.3389/fonc.2021.735338

**Published:** 2021-12-09

**Authors:** Tomas Bertok, Aniko Bertokova, Eduard Jane, Michal Hires, Juvissan Aguedo, Maria Potocarova, Ludovit Lukac, Alica Vikartovska, Peter Kasak, Lubor Borsig, Jan Tkac

**Affiliations:** ^1^ Institute of Chemistry, Slovak Academy of Sciences, Bratislava, Slovakia; ^2^ University Hospital Bratislava, Bratislava, Slovakia; ^3^ Center for Advanced Materials, Qatar University, Doha, Qatar; ^4^ Department of Physiology, University of Zurich, Zurich, Switzerland; ^5^ Comprehensive Cancer Center, Zurich, Switzerland

**Keywords:** colorectal cancer, lectin, microarray, glycosylation, biomarker

## Abstract

Colorectal cancer (CRC) is one of the most common types of cancer among men and women worldwide. Efforts are currently underway to find novel and more cancer-specific biomarkers that could be detected in a non-invasive way. The analysis of aberrant glycosylation of serum glycoproteins is a way to discover novel diagnostic and prognostic CRC biomarkers. The present study investigated a whole-serum glycome with a panel of 16 different lectins in search for age-independent and CRC-specific glycomarkers using receiver operating characteristic (ROC) curve analyses and glycan heat matrices. Glycosylation changes present in the whole serum were identified, which could lead to the discovery of novel biomarkers for CRC diagnostics. In particular, the change in the bisecting glycans (recognized by *Phaseolus vulgaris* erythroagglutinin) had the highest discrimination potential for CRC diagnostics in combination with human L selectin providing area under the ROC curve (AUC) of 0.989 (95% CI 0.950–1.000), specificity of 1.000, sensitivity of 0.900, and accuracy of 0.960. We also implemented novel tools for identification of lectins with strong discrimination power.

**Graphical Abstract:**
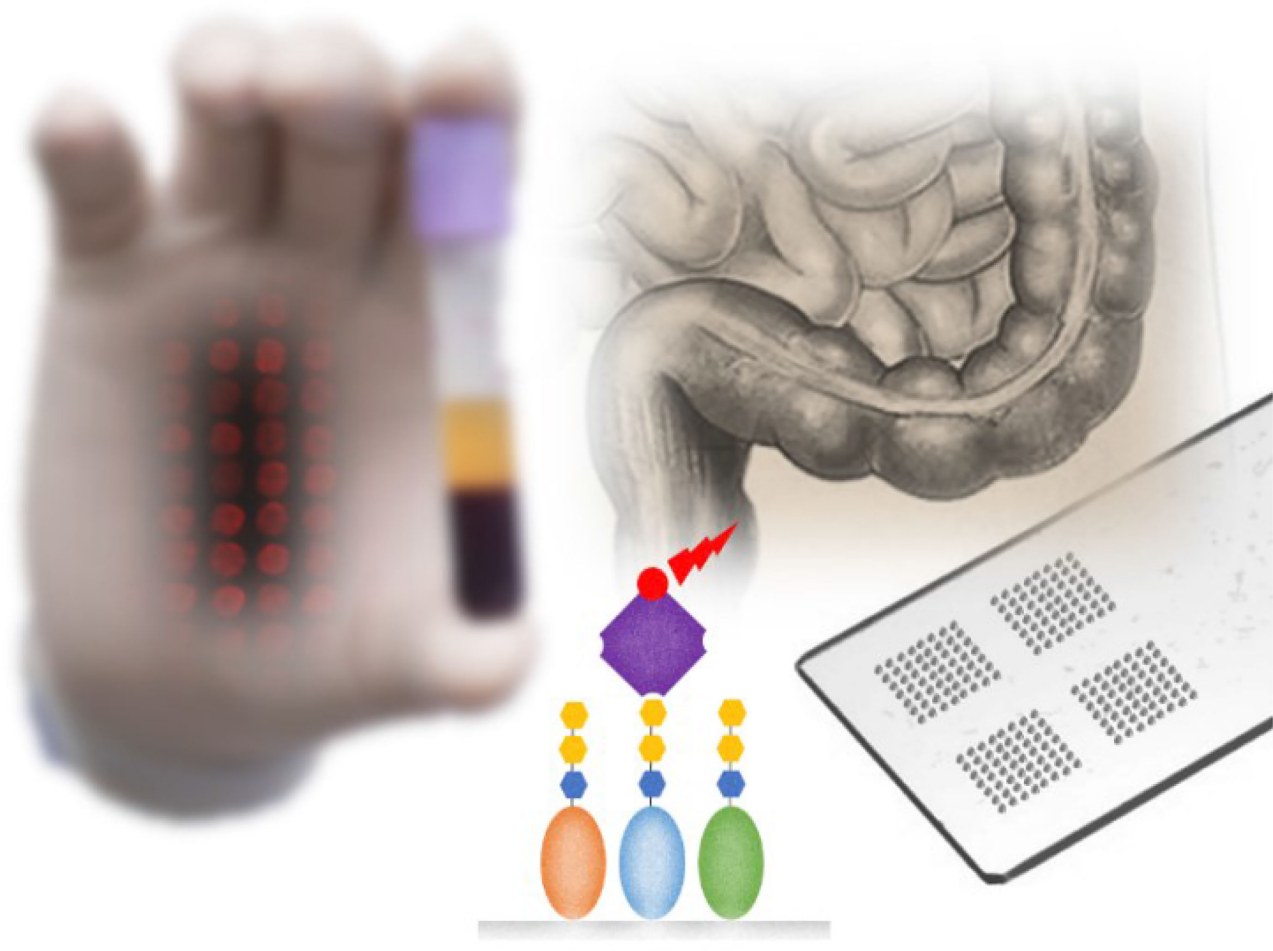


## Introduction

Cancer is regarded as the most important obstacle to increasing life expectancy in the 21st century. In both sexes, colorectal cancer (CRC) is one of the leading cancers, with incidence of ~1.8 M and mortality of 860,000 people annually (1), along with lung and prostate/breast cancers (2, 3). Moreover, it is estimated that, by 2030, there will be 2.2 M new CRC cases with an associated 1.1 M deaths (4, 5). Incidence increases rapidly with higher age and western lifestyle, while early diagnosis of localized tumors using sigmoidoscopy/colonoscopy with polypectomy increases the 5-year survival rate up to 90% (6). Point mutations in specific genes result in sporadic CRC in around 70% of all cases, while there is also a familial and inherited aspect behind CRC development and progression (7). A cancer biomarker is defined as a tumor characteristic that can be objectively measured and associated with the pathogenic process. Biomarkers are used for diagnostic, prognostic, predictive, and therapeutic purposes (8). In some cases, risk/predisposition biomarkers are used to identify people at significant risk of developing a disease. In addition to tissue biomarkers, such as cytokeratins, cadherin 17, or upregulation of telomerase expression (9), stool and blood biomarkers are of importance due to the less invasive collection of the sample.

CRC screening is performed using colonoscopy, flexible sigmoidoscopy, fecal occult blood testing, fecal DNA testing and by measuring the blood level of the carcinoembryonic antigen (CEA) (1, 10). Out of all these methods, colonoscopy is the gold standard due to the high sensitivity of the method, but the examination is uncomfortable for the patient and the accuracy of the examination depends on the skill level and experience of the operator (1). Although CEA is used for CRC diagnostics in combination with other examination methods, due to its low sensitivity and specificity, this biomarker is usually used in monitoring the effectiveness of the treatment and CRC recurrence (1). Additionally, CEA is also produced by other tissues in the human body, such as the breasts and pancreas (11), or is elevated under normal conditions, e.g., in smokers (12, 13). Moreover, besides CEA, other CRC biomarkers could be used in clinical practice with drawbacks similar to that of CEA (10, 11) and thus cannot be used in CRC screening but rather in monitoring the disease progression or CRC prognosis (9). Hence, there is a continuing need for biomarkers for early-stage CRC diagnosis, which can be detected in blood, serum, or plasma (liquid biopsy) (14, 15). Liquid biopsy-based assays were not yet recommended for CRC screening in the 2018 Guideline Updates from the American Cancer Society (16), but such tests may have a greater role in CRC screening in the future (16).

Very recently, novel blood-based biomarkers have been discovered, such as circulating tumor cells, circulating cell-free DNA, noncoding RNAs and microRNAs, and extracellular vesicles (mainly exosomes and oncosomes) (17). Besides several alternative CRC biomarkers, exosomes are regarded as a rich source of CRC biomarkers (proteins, glycoproteins, and various forms of RNAs), which can be effectively used for CRC diagnostics and CRC prognosis, as summarized in recent review papers (1, 18). Proteins expressed by exosomes were proven effective for the identification of CRC patients resistant to drug treatment or CRC patients with metastases (19).

A novel method for the discovery of CRC biomarkers is to identify glycosylation changes associated with CRC. Below, we describe the application of reverse-phase lectin microarrays for the identification of changes in the serum glycome as potential CRC biomarkers. A fluorescent microarray in combination with lectins is frequently used in the discovery of novel glycan-based biomarkers (20–24). In order to identify only those glycosylation changes that are associated with CRC and not with aging, serum samples in this study were analyzed as two distinct matrices, i.e., as the age matrix [healthy young (hY) vs. healthy old (hO)] and the CRC matrix (hO vs. CRC). Only those changes that are associated with the CRC matrix and not with the age matrix are deemed to be prospective novel CRC biomarkers.

## Experimental

Serum samples were taken from 34 individuals (6 healthy individuals with no CRC confirmed and with no comorbidities diagnosed at that time with an average age of 33.0 ± 6.1 years (hY cohort), 10 individuals with no malignancy with an average age of 67.0 ± 8.6 years (hO cohort), and 18 colorectal cancer patients with histologically proven CRC with an average age of 73.0 ± 7.3 (CRC cohort)). The Ethics Committee of the Faculty of Medicine, Comenius University and University Hospital in Bratislava, Old Town Hospital, Bratislava, Slovakia, approved the use of the samples, and all participants signed an informed consent document prior to sample collection. The procedure was performed under the ethical guidelines of the last revision of the Helsinki Declaration. Untreated serum samples were taken during the morning fasted state using a gel and clot activator tube (Vacutest Kima, Piove di Sacco, Italy). After 30 min, the tubes were centrifuged at 25°C for 10 min at 2,500 g. The sera were transferred into sterile plastic vials and were stored in the form of aliquots at -80°C until use and used within 1 year.

### Chemicals

All common chemicals [e.g., buffer components, bovine serum albumin (BSA), etc.] were purchased from Sigma Aldrich (USA). All solutions were freshly prepared prior to experiments in 0.055 μS deionized water (DW) and filtered using 0.2-μm sterile filters. Biotin conjugation kits for the biotinylation of unconjugated lectins were purchased from Abcam (UK). Lectins RPL-Fuc1 and RPL-Sia2 were obtained from GlycoSelect (Ireland). HPyL was purchased from GlycoDiag (France). P-selectin (P sel), L-selectin (HL sel), and E-selectin (HE sel) were used in the form of chimera proteins fused to IgG1 tail and obtained from Prof. Borsig with details provided in the paper (25). All the other lectins used were purchased in their biotinylated form from Vector Labs (USA). Conjugate streptavidin-CF647 was provided by Biotium (USA).

### Reverse-Phase Lectin Microarrays

Reverse-phase lectin microarray experiments were performed with a phosphate buffer solution (PBS) (0.01 M, pH 7.4) as a printing buffer. Spotting temperature was set at 10°C and humidity at 60%. Subsequently, the slide was placed in a humidity chamber for 1 h at ambient temperature (AT) with humidity of 80%–90%, blocked using a blocking buffer at ambient temperature for 1 h and with slow shaking, rinsed under a gentle stream of printing buffer in a Petri dish, and drained. For blocking purposes, 70 μl of 3% bovine serum albumin (BSA) was used, as we observed lower background fluorescence intensity than with a Carbo-free blocking solution (Vector Labs, USA) for some of the lectins used. Samples diluted 50× were spotted in two different wells in triplicates using SpotBot3 Microarray Protein edition (Arrayit, USA) on epoxide-coated slides Nexterion E (Schott, Germany) using a previously optimized protocol. Subsequently, after spotting and blocking the slides (1 h at AT with shaking), 70 μl of biotinylated lectins (c = 5 μg/ml in PBS) was added and incubated at AT for 1 h. The slides were washed gently three times with PBS, and then 70 μl of streptavidin-CF647 conjugate (c = 0.1 μg/ml in PBS) was added for 15 min. After a washing step and additional wash with DW, fluorescence intensity was read at 635 nm using an InnoScan microarray reader (Arrayit, USA). The signal evaluated and ascribed to individual samples using Mapix software was an average value of at least three spots after background fluorescence subtraction.

### Data Evaluation

All computations were performed using R software (version 3.6.3) (26) with CARET (Classification and Regression Training) and GLM packages (27). Receiver operating characteristic (ROC) curve, sensitivities, and specificities were estimated using the pROC package (28). The confidence intervals for area under the ROC curve (AUC) were computed by the bootstrap method with 2,000 stratified bootstrap replicates (29). Glycan heat matrices were prepared using OriginPro 2020.

Net reclassification improvement (NRI) and integrated discrimination improvement (IDI) were calculated according to Pencina et al. (30) using R package Hmics (https://CRAN.R-project.org/package=Hmisc). We computed continuous [category-free, NRI (>0)] NRI according to Pencina et al. (31). According to Pencina et al. (32), if NRI (>0) >0.6, there is strong discrimination, if NRI (>0) ~0.4, it is intermediate, and for NRI (>0) <0.2, discrimination between the models is considered weak. The heat maps are the NRI (>0) values. Only a combination of two lectins was considered in the evaluation. In the NRI (>0) and IDI evaluation, two models were compared. In our case, the old model of a single lectin was used and the updated model is the combination of two lectins.

The multicollinearity was tested using variance inflation factor (VIF) using R package car (33) with a VIF value above 10 indicating a multicollinearity problem (34).

## Results and Discussion

**Figure 1:**
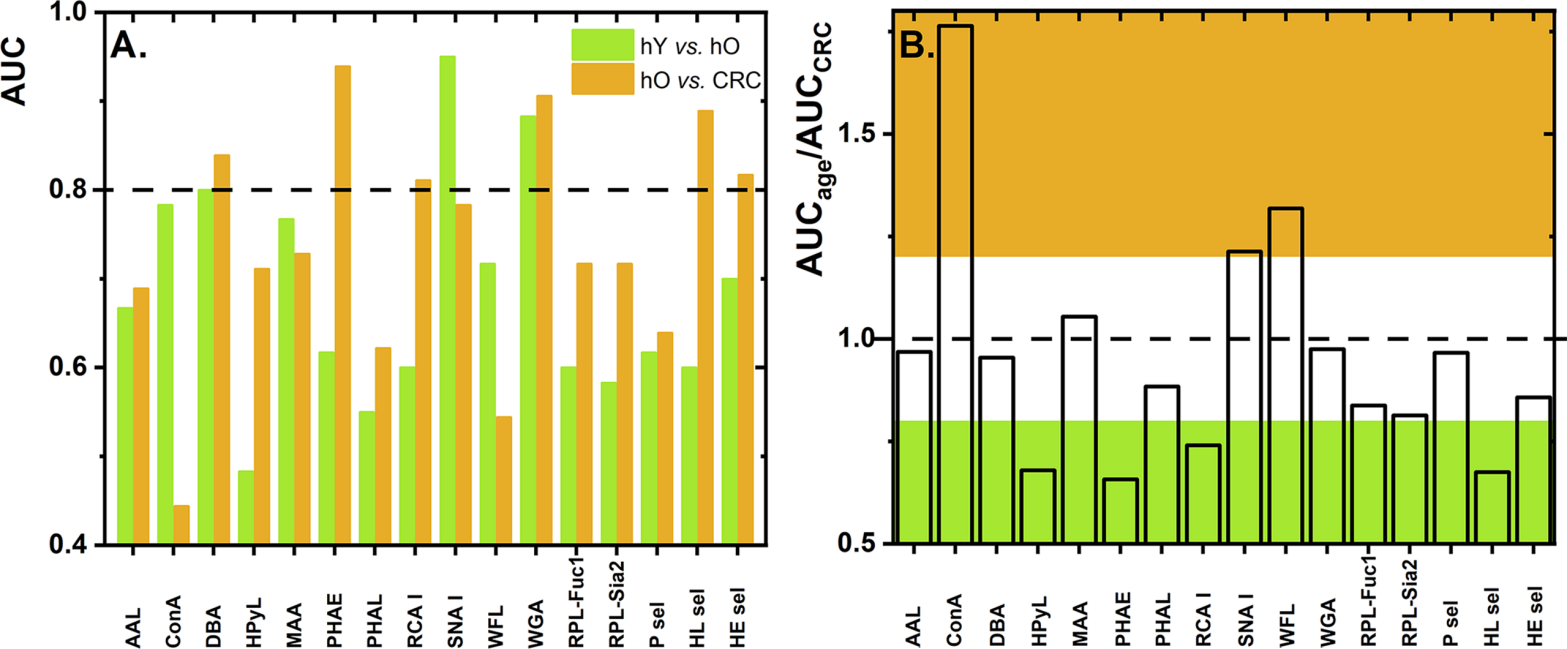
Values of AUC for discrimination of the age matrix [healthy young (hY) vs. healthy old (hO) individuals] and the CRC matrix [hO vs. CRC patients] using single lectins **(A)**. The ratio of AUC_age_ vs. AUC_CRC_ as determined for single lectins with the ratio below 0.8 is shown in green and the ratio above 1.2 is shown in brown **(B)**. Standard deviation (SD) values are not shown for the better visual clarity of both figures.

**Figure 2:**
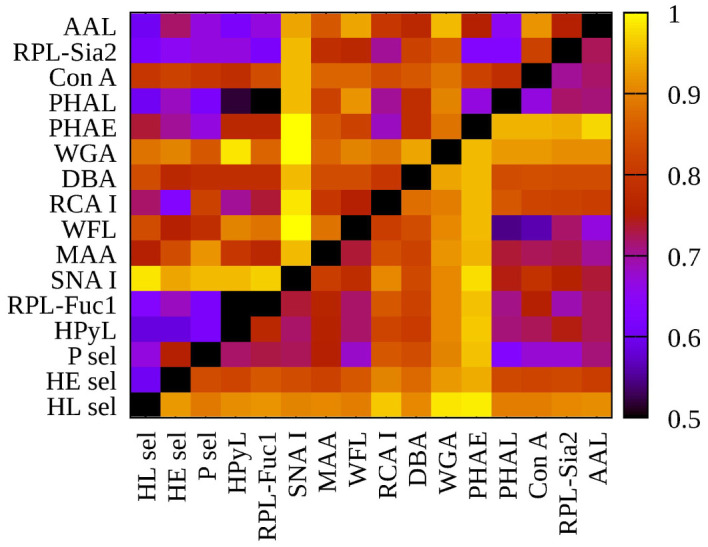
Heat map generated using 16 lectins and their performance according to AUC value for two submatrices, i.e., hY vs. hO (left upper triangle) and hO vs. CRC (right lower triangle).

Numerous studies have described glycosylation changes to be strongly associated with age (35–38). To clearly identify which glycan changes are strongly associated with age, we evaluated two healthy cohorts, i.e., hY (33.0 *±* 6.1 years old) and hO (67.0 *±* 8.6 years old). This discrimination is provided in the form of column graphs (**Figure 1**) for single lectins and a heat map as a submatrix 1 (hY vs. hO) in **Figure 2** for the combination of two lectins.

To evaluate glycosylation changes associated only with CRC, we also evaluated the CRC matrix, i.e., hO (67.0 *±* 8.6 years old) vs. CRC patients (73.0 *±* 7.3 years old). The results are shown in **Figure 1** for single lectins and **Figure 2** as a heat matrix for double lectins.

### Single Lectins

**Figure 1** shows the AUC values obtained when discrimination hY vs. hO (the age matrix) and hO vs. CRC (the CRC matrix) was investigated. More details of the clinical performance of single lectins to discriminate the age matrix vs. the CRC matrix are given in **Table S1**.

#### Weak Discrimination Power for Age Matrix vs. Colorectal Cancer Matrix

Discrimination hY vs. hO (the age matrix) and hO vs. CRC (the CRC matrix) for single lectins is shown in **Figures 1A, B**. The ratio AUC_age_/AUC_CRC_ was introduced here to investigate whether a single lectin has a discrimination power for the age matrix (high AUC_age_/AUC_CRC_ with a value higher than 1.2), a discrimination power for the CRC matrix (low AUC_age_/AUC_CRC_ with a value lower than 0.8), or only a moderate discrimination potential (AUC_age_/AUC_CRC_ within values 0.8–1.2). There are several lectins (i.e., AAL, DBA, MAA, PHAL, WGA, RPL-Fuc1, RPL-Sia2, P sel, and HE sel) with a really minor difference in the ratio AUC_age_/AUC_CRC_ (i.e., in the range of 0.8–1.2) (**Table S1** and **Figure 1B**). This means that such lectins can discriminate hY vs. hO and hO vs. CRC with similar performance, i.e., similar AUC. Hence, such lectins have only limited potential for CRC diagnostics.

#### Strong Discrimination Power for the Age Matrix

There are three lectins that can be used in selective discrimination of the age matrix over the CRC matrix, such as Concanavalin A (ConA) (AUC_age_/AUC_CRC_ = 1.76); WFL (AUC_age_/AUC_CRC_ = 1.32), and SNA I (AUC_age_/AUC_CRC_ = 1.21) (**Figure 1B** and **Table S1**). Serum proteins are present in the blood in the following descending order: IgG (40.4%), IgA (9.0%), serotransferrin (8.5%), IgM (5.0%), haptoglobin (4.5%), α-1-acid glycoprotein (2.6%), ceruloplasmin (1.2%), β-2-glycoprotein I (0.7%), apolipoprotein B-100 (0.5%), apolipoprotein D (0.3%), and IgD (0.1%) (39). Hence, the most abundant protein in the blood is IgG, which might be the most likely carrier of glycans determined in this study. Several studies describe that, at early adulthood, there is a high abundance of digalactosylated IgG forms and that, with increasing age, a decrease in galactosylation and sialylation can be observed (37). Thus, the SNA I lectin should be a positive discriminator when used for the age matrix (i.e., hY vs. hO), as was experimentally confirmed in this study (**Figure 1A**). In the other study using the reverse-phase lectin microarray, it was found that the SNA I binding to transferrin isolated from human serum decreased with age (40), suggesting that there might be other protein carriers besides IgG-carrying glycans recognized by SNA I.

Another lectin, which is more suitable for discrimination of the age matrix rather than for discrimination of the CRC matrix, is WFL lectin. Since WFL especially recognizes GalNAc and LacdiNAc glycan structures, this might indicate that such glycan structures are present on other proteins in the blood and not on IgG, which has a rather conserved biantennary structure.

The significantly increased AUC value for ConA to discriminate the age matrix might indicate a decrease in the overall glycosylation of proteins present in the serum with age or a decrease in oligo-mannose-containing glycans. Changes in the glycosylation of proteins can also be an indicator of other processes in the body, including inflammation and other diseases (37).

There is one negative correlation for discriminating hY vs. hO, i.e., application of HPyL. This lectin is produced by GlycoDiag with the source of Human Polyomavirus 9 VP1 (**Table 1**). This lectin preferentially binds short, linear glycan sequences terminating in *N*-glycolylneuraminic acid (Neu5Gc) (46). Neu5Gc is a non-human derivative of sialic acid delivered to the human body in the form of red meat. The only explanation for the result obtained using HPyL is that the serum of older healthy individuals contains a larger amount of Neu5Gc as a result of a higher intake of red meat for elderly people.

**Table 1:** Lectin specificity for the lectins applied in this study.

Lectins	Source	Glycan specificity
AAL	*Aleuria aurantia* mushrooms	Fucα6GlcNAc (core Fuc), Fucα3(Galβ4)GlcNAc (Le^x^)
RPL-Fuc1	*Aspergillus fumigatus* lectin	Fucα3GlcNAc, Fucα4GlcNAc, Le^a^, Le^b^, Le^x^, Le^y^
PHAE (erythroagglutinin)	*Phaseolus vulgaris* seeds	*N*-glycans with outer Gal and bisecting GlcNAc
PHAL (leukoagglutinin)	*Phaseolus vulgaris* seeds	tri/tetra-antennary *N*-glycans
ConA	*Canavalia ensiformis* bean seeds	αMan, αGlc; high-Man; Manα6(Manα3)Man; Manα6Man; Manα3Man
DBA	*Dolichos biflorus* seeds	αGalNAc; terminal GalNAc; GalNAcα3GalNAc
WFL	*Wisteria floribunda* lectin	GalNAc, LacdiNAc
WGA	*Triticum vulgaris*	(GlcNAcβ4)_n_, Neu5Ac; poly(*N*-acetyllactosamine)
RCA I	*Ricinus communis* seeds	Gal; Galβ4GlcNAc
MAA	*Maackia amurensis* seeds	Neu5Acα3Galβ4GalNAc; 3-*O*-Suα3Galβ4GalNAc; sT antigen
P sel	human	sLe^x^ (Neu5Acα3Galβ4(Fucα3)GlcNAc); sLe^a^ (Neu5Acα3Galβ4(Fucα4)GlcNAc); sulfo groups
RPL-Sia2	*Streptococcus gordonii* M99	Neu5Acα3 on *O*-glycans; Neu5Acα3Galβ3GalNAc (*O*-glycans) > Neu5Acα2-3Galβ4Glc (*N*-glycans)
SNA I	*Sambucus nigra* bark	Neu5Acα6Galβ4GalNAc; 6-*O*-Suα3Galβ4GalNAc; sTn antigen
HPyL	Human Polyomavirus 9 VP1	Neu5Gcα3Galβ4GlcNAc; Neu5Gcα3Galβ4Glc; Neu5Acα3Galβ4GlcNAc
HE sel	human	sLe^x^ (Neu5Acα3Galβ4(Fucα3)GlcNAc)
HL sel	human	6-O-Su sLe^x^ i.e. Neu5Acα3Galβ4(Fucα3)(Su6)GlcNAc); Neu5Acα3Galβ4(Fucα1-3)(Su6)Glc); sulfo groups

Table adapted from our previous study (41) with data taken from Vector Laboratories and GlycoDiag leaflets and from Refs (22, 25, 42–46).

Another glycan change referred to in the literature is a decrease in the level of Neu5Ac attached to Gal *via* α2,3-linkage (38) with age, which was also observed in this work with an AUC value of 0.767 for the MAA lectin for the age matrix.

#### Strong Discrimination Power for the Colorectal Cancer Matrix

There are, however, several lectins with a good discrimination power for the CRC matrix rather than for the age matrix, which is a feature applicable to CRC diagnostics, i.e., PHAE (AUC_age_/AUC_CRC_ = 0.66), HPyL (AUC_age_/AUC_CRC_ = 0.68), HL sel (AUC_age_/AUC_CRC_ = 0.68), and RCA I (AUC_age_/AUC_CRC_ = 0.74).

Three out of those four lectins have AUC exceeding a value of 0.8, i.e., PHAE, RCA I, and HL sel, with a potential application for CRC diagnostics.

Changes in the glycosylation of proteins in the sera of CRC patients were recently extensively reviewed (47). The CRC patients were observed to have a decreased occurrence in bisecting *N*-acetyl-lactosamine (LacNAc) structures and an increase in truncated paucimannosidic (high-mannose) structures for *N*-glycans and a decrease in Core 3 and Core 4 structures for *O*-glycans (47). Moreover, a decrease in extended *O*-glycans was observed with CRC development and progression (48).

PHAE lectin specifically recognizes bisecting glycans (49), and the present study showed that CRC patients possessed a lower amount of bisecting *N*-glycans, which is in agreement with previous conclusions (47). Moreover, a lectin microarray study focused on the analysis of glycosylation changes of transferrin isolated from human serum showed a decreased response toward transferrin’s glycans recognized by PHAE (40). This might indicate that transferrin could also be a potential carrier of the glycans recognized by PHAE in our study, since transferrin is an abundant protein in the serum (8.5% of total serum proteins).

The RCA I-recognizing lectin indicates that Gal and Galβ4GlcNAc-containing glycans are present at a lower level in CRC patients in comparison with healthy individuals. This finding is in agreement with the results from a lectin microarray study focused on a change of transferrin’s glycans related to CRC (40). This might indicate transferrin to be a potential carrier of glycans recognized by RCA I.

It is remarkable that HPyL-recognizing Neu5Gc in our study has a good negative discrimination power (i.e., increased level for healthy individuals in comparison with the CRC patients) (**Figure 1A**), since there is quite a strong correlation between red meat uptake and CRC (50, 51). It is well known that red meat contains sialic acid in the form of Neu5Gc, and it was recently shown that the level of Neu5Gc is higher in CRC patients than in healthy individuals (52). However, there is an explanation for this apparent discrepancy. We found an increased level of Neu5Gc in the healthy old cohort in comparison with the healthy young cohort. After CRC diagnosis, such CRC patients might be on a low red meat diet, which might result in a reduced amount of Neu5Gc in the CRC patients.

From all three selectins used in the study, it was found that HL sel had a strong discrimination potential for the CRC matrix (**Figure 1A**). A lectin-binding preference (**Table 1**) shows that HL sel has quite a strong binding preference for sulfated glycans. The results show a greater abundance of sulfated glycans in the healthy old cohort than in the CRC patient cohort. It is not yet clear what kind of glycosylation change to expect regarding the acetylation and sulfation of glycans during CRC development and/or progression (47).

The results related to discrimination of the ConA lectin are in agreement with the literature (40, 47) showing an increased level of mannosylated (truncated paucimannosidic) *N*-glycan structures associated with CRC.

### Combination of Two Lectins

Evaluation of double biomarkers for discrimination of the age matrix and the CRC matrix resulted in 120 combinations for each matrix, i.e., in total 240 combinations (**Table S1** and **Figure 2**). Accordingly, in the following section, we discuss only the double lectin combinations with lectins identified in the previous sections as lectins showing the best discrimination performance for the particular matrix.

Since SNA I showed AUC above 0.8 (i.e., 0.95) and the ratio of AUC_age_/AUC_CRC_ above 1.2 (i.e., 1.21), only the double lectin biomarkers with SNA I for the age matrix will be evaluated here. As for the CRC matrix, only the double lectin combinations with PHAE (AUC = 0.939, AUC_age_/AUC_CRC_ = 0.657), RCA I (AUC = 0.811; AUC_age_/AUC_CRC_ = 0.74), or HL sel (AUC = 0.889, AUC_age_/AUC_CRC_ = 0.675) will be evaluated.

#### Strong Discrimination Power for the Age Matrix

From all the 15 combinations of double lectin biomarkers with SNA I in **Table 2**, there is a selection of only those combinations with an AUC value that exceeds that of the AUC value of the single SNA I lectin, i.e., the AUC value of 0.95. Thus, in **Table 2**, clinical performance parameters are shown for two such combinations having a significant power to discriminate between the age matrix over the CRC matrix, as judging from the AUC_age_/AUC_CRC_ ratio above 1.2, i.e., SNA I+RPL-Fuc1 (1.319) and SNA I+WFL (1.285). This means that two such combinations of lectins have the potential to be used in the selective discrimination of age. A literature review identified a paper where the ratio of two biantennary glycans present in the serum, i.e., G0F/G2F (G0F denotes an agalactosylated bianntennary glycan with core fucose, and G2F denotes a digalactosylated biannennary glycan with core fucose) increased with age (53). This ratio was then applied as a GlycoAgeTest for the detection of biological age (53). The other study confirmed a decrease in G2F and an increase in G0F of IgG (54) in agreement with the previous study (53). Moreover, Pučić et al. (54) identified a decrease in the IgG sialylation with aging. The results in the present study indicate that SNA I can detect the change in the sialylation of *N*-glycans associated with age (**Figure 1**), which confirms the observation in the study by Pučić et al. (54), and that most probably the protein carrier associated with such glycosylation changes is IgG. For example, changes in glycosylation including galactosylation, sialylation, and bisecting glycans were used in the calculation of the GlycanAge index (55).

**Table 2:** Clinical performance characteristics of double lectins for the age matrix with the best combination showed in red.

Lectins	AUC	AUC left	AUC right	Spec	Sens	Acc	AUC_age_/AUC_CRC_
SNA I + WFL	1	1	1	1	1	1	1.285
SNA I + RPL-Fuc1	0.967	0.867	1	0.833	1	0.896	1.319

AUC, area under the ROC (receiver operating characteristic) curve; AUC left, a lower interval for the 95% confidence interval for AUC value; AUC right, an upper interval for the 95% confidence interval for AUC value; Spec, specificity; Sens, sensitivity; Acc, accuracy. Only lectin combinations with a ratio of AUC_age_/AUC_CRC_ exceeding 1.2 are shown.

**Figure 3:**
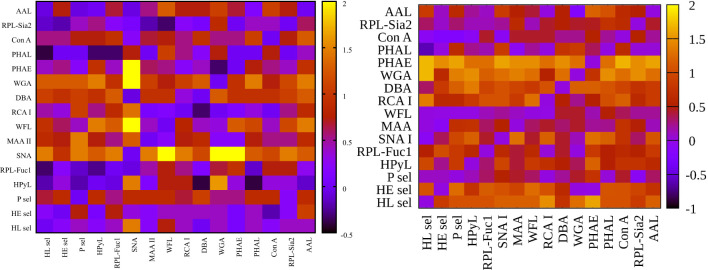
Heat maps for net reclassification improvement (NRI) (>0) analysis showing hY vs. hO (left) and hO vs. CRC (right).

**Figure 4:**
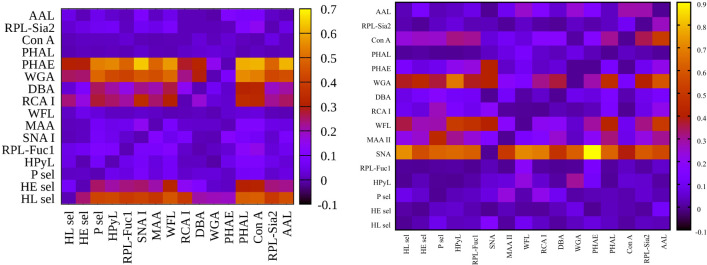
Heat maps for integrated discrimination improvement (IDI) analysis showing hY vs. hO (left) and hO vs. CRC (right).

**Figure 5:**
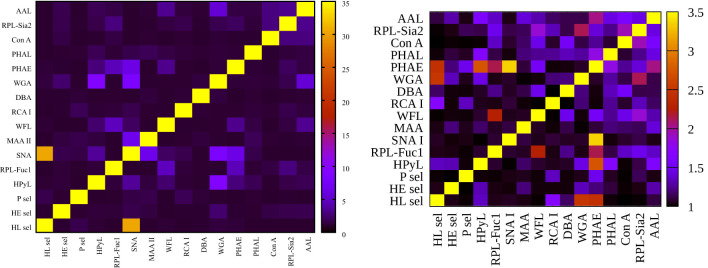
Heat maps for variance inflation factor (VIF) analysis showing hY vs. hO (left) and hO vs. CRC (right).

Our results indicate that a combination of SNA I with two other lectins, i.e., RPL-Fuc1 and WFL, has the potential to effectively discriminate age, in particular a decreased fucosylation (recognized by RPL-Fuc1 but not by AAL) and a decrease in the level of GalNAc- and LacdiNAc-containing glycans (recognized by WFL). It is obvious that there is an age-related change in the glycosylation of IgG and other serological proteins in the process called inflammaging (chronic and low-grade inflammation progressing with age) (56). **Table 2** shows that a combination of lectins SNA I + WFL displayed the highest accuracy in discriminating the age matrix, as was confirmed by an additional statistical evaluation (**Figure 3** **left**; **Figure 4** **left**). Furthermore, the statistical evaluation showed CIF well below a threshold value of 10 for each lectin combination including SNA I + WFL (**Figure 5** **left**), not indicating a multicollinearity problem. The only exception is the combination of SNA I with HL sel with a VUF value of 30.4, which can be explained by the fact that both lectins recognize the same glycan epitope (i.e., α2,6-linked sialic acid).

**Figure 3** **left** (hY vs. hO) shows a strong intensity in the horizontal for lectin SNA and slightly less intensive for lectin WGA. These lectins provide strong discrimination in case of hY vs. hO (only these two lectins have AUC over 0.8 in single lectin evaluation as shown in **Figure 1**). **Figure 3** **right** (hO vs. CRC) shows a high intensity for the lectins DBA, PHAE, RCA I, WGA, and selectins, which reached AUC above the value of 0.8 in a single lectin evaluation. Lectin PHA-E has the highest AUC, and PHAE lectin also shows a horizontal row with the highest values of NRI. Clearly, the NRI values confirm the results presented in **Figure 1**, which are the best lectins to improve discrimination hY vs. hO or hO vs. CRC, respectively.

The IDI values show similar conclusions (**Figure 4**) as made for the NRI values (**Figure 3**). In case of the hY vs. hO, the highest values of IDI were observed for lectins SNA and WGA (**Figure 4** **left**), while for the discrimination of hO vs. CRC, the following lectins showed high values: PHAE, WGA, DBA, RCA I, and selectins (**Figure 4** **right**).

We can conclude that it is easier to identify lectins with strong discrimination power hY vs. hO or hO vs. CRC using the IDI matrix (**Figure 4**) compared to the NRI matrix (**Figure 3**).

#### Strong Discrimination Power for the Colorectal Cancer Matrix

In the CRC matrix, only the double lectin combinations with PHAE (AUC = 0.939, AUC_age_/AUC_CRC_ = 0.657), RCA I (AUC = 0.811; AUC_age_/AUC_CRC_ = 0.74), or HL sel (AUC = 0.889, AUC_age_/AUC_CRC_ = 0.675) are evaluated.

All combinations of PHAE with any other lectin shown in **Table 3** provide AUC higher than for a single PHAE (AUC of 0.939) with the discrimination AUC_age_/AUC_CRC_ ratio below 0.8, as was confirmed for most combinations of PHAE with other lectins by an additional statistical evaluation (**Figure 3** **right** and **Figure 4** **right**). The lectin combination PHAE + HL sel provided the highest discrimination accuracy based on the AUC value (0.989).

**Table 3:** Clinical performance characteristics of double lectins for the CRC matrix with the best combinations showed in red.

Lectins	AUC	AUC left	AUC right	Spec	Sens	Acc	AUC_age_/AUC_CRC_
PHAE + AAL	0.972	0.9	1	0.944	0.9	0.929	0.772
PHAE + HPyL	0.961	0.883	1	0.833	1	0.893	0.798
PHAE + PHAL	0.944	0.844	1	0.778	1	0.857	0.707
PHAE + RCA I	0.95	0.856	1	0.944	0.9	0.929	0.719
PHAE + RPL-Sia2	0.939	0.828	1	0.833	1	0.893	0.674
PHAE + P sel	0.956	0.861	1	0.944	0.9	0.929	0.698
PHAE + HL sel	0.989	0.95	1	1	0.9	0.964	0.741
RCA I + PHAE	0.95	0.856	1	0.944	0.9	0.929	0.719
RCA I + HL sel	0.961	0.883	1	1	0.8	0.929	0.746
RCA I + HE sel	0.9	0.75	1	0.944	0.8	0.893	0.703
HL sel + AAL	0.911	0.778	1	0.833	0.9	0.857	0.659
HL sel + HPyL	0.911	0.778	1	1	0.7	0.893	0.64
HL sel + PHAE	0.989	0.95	1	1	0.9	0.964	0.741
HL sel + PHAL	0.894	0.761	0.994	0.944	0.7	0.857	0.671
HL sel + RCA I	0.961	0.883	1	1	0.8	0.929	0.746
HL sel + RPL-Fuc1	0.917	0.789	1	1	0.7	0.893	0.69
HL sel + RPL-Sia2	0.906	0.761	0.994	0.944	0.7	0.857	0.681
HL sel + P sel	0.889	0.728	1	1	0.7	0.893	0.75
HL sel + HE sel	0.922	0.8	0.994	0.833	0.9	0.857	0.651

see **Table 2**. Only lectin combinations with the ratio of AUC_age_/AUC_CRC_ below 0.8 are shown.

Three combinations of RCA I with other lectins provided significantly higher AUC than a single RCA I lectin with a higher discrimination ratio AUC_age_/AUC_CRC_ (**Table 3**). A combination of RCA I + HL sel provided the highest discrimination accuracy for the CRC matrix based on AUC value (0.961).

With regard to double lectin combinations with HL sel, a high discrimination power is a result of the combination with RCA I and PHAE lectins, which is the anticipated outcome, since RCA I and PHAE lectins have a strong discrimination power for the CRC matrix as single lectins. The best discrimination power based on the AUC value (0.989) for the CRC matrix was obtained using the combination of HL sel + PHAE (**Table 3** **)**. Furthermore, statistical evaluation showed CIF well below a threshold value of 10 for every lectin combination (**Figure 5** **right**) with no multicollinearity problem.

From **Table 3**, it can be concluded that the best double lectin biomarkers are a combination of PHAE + HL sel with AUC of 0.989 (**Table 3**) with high discrimination power for the CRC matrix (AUC_age_/AUC_CRC_ = 0.741).

Several studies describe the use of glycan analysis for CRC diagnostics. All the papers discussed below are instrument-based approaches applied to glycan analysis.

Detection of IgG Fc *N*-glycopeptides by nanomaterial enrichment with subsequent MALDI-TOF mass spectrometry (MS) analysis of plasma samples from 46 CRC patients and 67 healthy individuals was evaluated in the form of an ROC curve. Machine learning algorithm using analysis of 11 *N*-glycopeptides was able to distinguish CRC patients from healthy controls with the average AUC value of the ROC of 0.893 (57).

An analysis of glycopeptides isolated from the serum of 80 CRC patients and 50 healthy individuals using an instrument-based approach revealed that especially leucine-rich α-2-glycoprotein with fucosylated triantennary *N*-glycan was a prospective diagnostic CRC biomarker (with high AUC of 0.86, sensitivity of 0.80, and specificity of 0.74) especially in combination with CEA (12).

Analysis of the ratio of two glycans with an AUC value above 0.95 was able to discriminate between CRC patients (n = 20) and healthy individuals (n = 20) (58).

The changed glycosylation of IgG isolated from the serum of CRC patients (n = 36), people with benign disease (n = 23), and a healthy control (n = 19) was also studied (59). The results indicate that glycans can only moderately discriminate the benign disease from the early-stage CRC (AUC = 0.75), and the early-stage CRC vs. the late-stage CRC (AUC = 0.75) with significant discrimination observed only between the late-stage CRC vs. benign disease (AUC = 0.85) (59). Accordingly, such an approach is not suitable for early-stage CRC diagnostics.

Another study revealed the CRC to be associated with a decrease in IgG galactosylation and IgG sialylation and an increase in core-fucosylation of neutral glycans with a concurrent decrease in the core-fucosylation of sialylated glycans. Glycan analysis rendered it possible to discriminate CRC patients from the control cohort with the AUC value of 0.755 (60).

Significant differences in the total serum *N*-glycome between CRC patients and the control cohort were used for CRC diagnostics (61). The authors observed an increased branching and sialylation for CRC patients, while the control cohort showed predominantly biantennary glycans. Glycan analysis could discriminate CRC patients from the control cohort with the AUC value of 0.81, sensitivity of 0.72, and specificity of 0.79. The 5-year survival rate largely varied between CRC patients with an altered serum *N*-glycome (46%) and an *N*-glycome similar to controls (87%). Importantly, the total serum *N*-glycome showed a prognostic value beyond age and stage (61).

It is worth mentioning that instrument-based methods are regarded as low-throughput and high-cost approaches to glycan analysis (12). The present study is the first using a reverse-phase lectin microarray for evaluation of the potential of glycans for CRC diagnostics with important clinical performance parameters obtained. It is difficult to identify protein carriers carrying aberrant glycans detected by lectins in this study, but some clues could be found in our recent study (41). A clear advantage of the approach presented here is that the most promising lectins (PHAE, RCA I, and HL sel) having a diagnostic potential for CRC could be used in the selective enrichment of serum proteins. In that case, lectin columns (PHAE, RCA I, and HL sel) will be used for the fractionation of serum proteins with subsequent protein identification using MS techniques. Hence, it would be possible to identify serum glycoproteins carrying aberrant glycans recognized by PHAE, RCA I, or HL sel, which could be more reliable CRC biomarkers based on glycans than, for example, CEA.

Such a lectin combination has the potential for use in the selective fractionation of serum proteins using lectin columns or lectins attached to magnetic particles. In that way, we will pre-enrich glycoproteins with specific aberrant glycosylation profiles. Then, we will identify such glycoproteins by MS using peptide mapping. Finally, such identified glycoproteins can be tested as potential novel glycan-based biomarkers for CRC diagnostics using specific glycoprofiling, i.e., application of an antibody selective against such a protein for selective capture from serum samples with subsequent glycoprofiling using lectins in a sandwich configuration (antibody/glycoprotein/lectin) using ELISA format of analysis, which is fully compatible with clinical practice (62, 63).

## Conclusions

This study used novel recombinant lectins and lectins of human origin in combination with reverse-phase lectin microarrays to investigate glycosylation changes in the whole serum associated with age and with CRC. To the best of our knowledge, the lectin-recognizing Neu5Gc (HPyL) was used for the first time on reverse-phase lectin microarrays. The study identified lectins (SNA I + WFL) that can be specifically used for the discrimination of age and thus for determining biological human age. Then, we identified the combination of lectins (PHAE + HL sel), which can detect only glycosylation changes associated with CRC and not with age.

## Data Availability Statement

The original contributions presented in the study are included in the article/**Supplementary Material**. Further inquiries can be directed to the corresponding author.

## Ethics Statement

The studies involving human participants were reviewed and approved by the ethical committee of University Hospital in Bratislava. The patients/participants provided their written informed consent to participate in this study.

## Author Contributions

TB, AB, and JT contributed with concept formulation and the study design. TB, AB, MH, JA, and AV conducted experimental work. MP and LL provided samples and associated data and prepared the first draft of the article. TB and EJ performed statistical data evaluation and data presentation. TB, PK, LB, and JT wrote the article. All authors contributed to the article and approved the submitted version.

## Funding

The financial support received from VEGA 2/0130/20 and from the Slovak Research and Development Agency APVV 17-0300 is acknowledged. The authors would like to acknowledge the support received from the Ministry of Health of the Slovak Republic under the project registration number 2018/23-SAV-1. The authors wish to acknowledge the financial support received from the Innovative Training Network (No. 813120). This publication was jointly supported by Qatar University and Chemical Institute, Slovak Academy of Sciences-IRCC-2020-004. The finding achieved herein is solely the responsibility of the authors. The statements made herein are solely the responsibility of the authors. This publication was created with the support of the Operational Programme Integrated Infrastructure for the project: Centre for Biomedical Research-BIOMEDIRES-II stage, ITMS: 313011W428, co-financed by the European Regional Development Fund. 

## Conflict of Interest

The authors declare that the research was conducted in the absence of any commercial or financial relationships that could be construed as a potential conflict of interest.

## Publisher’s Note

All claims expressed in this article are solely those of the authors and do not necessarily represent those of their affiliated organizations, or those of the publisher, the editors and the reviewers. Any product that may be evaluated in this article, or claim that may be made by its manufacturer, is not guaranteed or endorsed by the publisher.
