# Supplementary material for: Identification of Whole-Serum Glycobiomarkers for Colorectal Carcinoma Using Reverse-Phase Lectin Microarray
Source: Front Oncol. 2021 Dec 9;11:735338. doi: 10.3389/fonc.2021.735338 (PMC8695905; doi:10.3389/fonc.2021.735338)
Supplement: Supplementary file 1 [file DataSheet_1.pdf]

hY vs. hO		AUC	AUC left	AUC right	Spec	Sens	Acc		hO vs. CRC		AUC	AUC left	AUC right	Spec	Sens	Acc		AUC_{age}/AUC_{CRC}
1	~ AAL	0.667	0.333	1	0.667	0.9	0.754		1	~ AAL	0.689	0.467	0.894	0.778	0.7	0.75		0.968
2	~ ConA	0.783	0.483	1	0.667	0.9	0.754		2	~ ConA	0.444	0.233	0.667	0.667	0.5	0.607		1.764
3	~ DBA	0.8	0.533	1	1	0.7	0.888		3	~ DBA	0.839	0.661	0.978	0.722	0.9	0.786		0.954
4	~ HPyL	0.483	0.183	0.783	0.667	0.5	0.604		4	~ HPyL	0.711	0.461	0.922	0.889	0.6	0.786		0.679
5	~ MAA	0.767	0.467	0.967	1	0.5	0.813		5	~ MAA	0.728	0.528	0.894	0.556	1	0.714		1.054
6	~ PHAE	0.617	0.267	0.933	0.5	0.9	0.65		6	~ PHAE	0.939	0.833	1	0.944	0.9	0.929		0.657
7	~ PHAL	0.55	0.267	0.833	0.833	0.5	0.708		7	~ PHAL	0.622	0.383	0.867	0.778	0.7	0.75		0.884
8	~ RCA I	0.6	0.3	0.867	1	0.4	0.775		8	~ RCA I	0.811	0.606	0.967	0.833	0.7	0.786		0.74
9	~ SNA I	0.95	0.833	1	1	0.8	0.925		9	~ SNA I	0.783	0.589	0.944	0.778	0.8	0.786		1.213
10	~ WFL	0.717	0.4	0.95	0.5	1	0.688		10	~ WFL	0.544	0.311	0.756	0.278	1	0.536		1.318
11	~ WGA	0.883	0.683	1	0.833	0.9	0.858		11	~ WGA	0.906	0.756	1	0.944	0.8	0.893		0.975
12	~ RPL-Fuc1	0.6	0.267	0.9	0.5	0.8	0.613		12	~ RPL-Fuc1	0.717	0.517	0.883	0.556	0.9	0.679		0.837
13	~ RPL-Sia2	0.583	0.25	0.867	0.667	0.6	0.642		13	~ RPL-Sia2	0.717	0.489	0.917	0.944	0.5	0.786		0.813
14	~ P sel	0.617	0.317	0.884	0.833	0.6	0.746		14	~ P sel	0.639	0.4	0.861	0.944	0.4	0.75		0.966
15	~ HL sel	0.6	0.3	0.867	0.833	0.5	0.708		15	~ HL sel	0.889	0.739	0.989	0.944	0.7	0.857		0.675
16	~ HE sel	0.7	0.433	0.933	1	0.5	0.813		16	~ HE sel	0.817	0.617	0.967	0.722	0.9	0.786		0.857
17	~ AAL + ConA	0.917	0.733	1	0.833	0.9	0.858		17	~ AAL + ConA	0.717	0.517	0.911	0.778	0.7	0.75		1.279
18	~ AAL + DBA	0.767	0.5	1	0.833	0.8	0.821		18	~ AAL + DBA	0.833	0.65	0.978	0.944	0.7	0.857		0.921
19	~ AAL + HPyL	0.617	0.267	0.917	0.667	0.7	0.679		19	~ AAL + HPyL	0.722	0.472	0.922	0.833	0.7	0.786		0.855
20	~ AAL + MAA	0.85	0.6	1	1	0.7	0.888		20	~ AAL + MAA	0.7	0.494	0.889	0.556	0.9	0.679		1.214
21	~ AAL + PHAE	0.75	0.467	0.95	1	0.5	0.813		21	~ AAL + PHAE	0.972	0.9	1	0.944	0.9	0.929		0.772
22	~ AAL + PHAL	0.65	0.3	1	0.667	0.9	0.754		22	~ AAL + PHAL	0.711	0.483	0.906	0.778	0.8	0.786		0.914
23	~ AAL + RCA I	0.8	0.517	1	0.833	0.8	0.821		23	~ AAL + RCA I	0.811	0.617	0.967	0.833	0.8	0.821		0.986
24	~ AAL + SNA I	0.933	0.783	1	1	0.8	0.925		24	~ AAL + SNA I	0.733	0.506	0.917	0.778	0.8	0.786		1.273
25	~ AAL + WFL	0.933	0.75	1	1	0.9	0.963		25	~ AAL + WFL	0.667	0.444	0.872	0.778	0.7	0.75		1.399
26	~ AAL + WGA	0.95	0.8	1	0.833	1	0.896		26	~ AAL + WGA	0.911	0.767	1	0.944	0.8	0.893		1.043
27	~ AAL + RPL-Fuc1	0.667	0.35	0.933	0.833	0.6	0.746		27	~ AAL + RPL-Fuc1	0.722	0.517	0.894	0.611	0.9	0.714		0.924
28	~ AAL + RPL-Sia2	0.75	0.433	1	0.667	0.9	0.754		28	~ AAL + RPL-Sia2	0.722	0.489	0.911	0.889	0.6	0.786		1.039
29	~ AAL + P sel	0.667	0.4	0.917	1	0.5	0.813		29	~ AAL + P sel	0.711	0.494	0.894	0.889	0.5	0.75		0.938
30	~ AAL + HL sel	0.6	0.3	0.883	0.667	0.6	0.642		30	~ AAL + HL sel	0.911	0.778	1	0.833	0.9	0.857		0.659
31	~ AAL + HE sel	0.717	0.433	0.983	0.833	0.7	0.783		31	~ AAL + HE sel	0.811	0.606	0.967	0.722	0.9	0.786		0.884
32	~ ConA + DBA	0.85	0.633	1	0.667	0.9	0.754		32	~ ConA + DBA	0.839	0.655	0.978	0.833	0.8	0.821		1.013
33	~ ConA + HPyL	0.783	0.466	1	0.667	1	0.792		33	~ ConA + HPyL	0.722	0.467	0.939	0.833	0.7	0.786		1.084
34	~ ConA + MAA	0.867	0.65	1	1	0.6	0.85		34	~ ConA + MAA	0.722	0.517	0.9	0.556	1	0.714		1.201
35	~ ConA + PHAE	0.817	0.567	1	0.667	0.9	0.754		35	~ ConA + PHAE	0.944	0.833	1	0.944	0.9	0.929		0.865

36	~ ConA + PHAL	0.783	0.5	1	0.833	0.7	0.783		36	~ ConA + PHAL	0.667	0.411	0.9	0.833	0.7	0.786		1.174
37	~ ConA + RCA I	0.833	0.567	1	0.667	0.9	0.754		37	~ ConA + RCA I	0.822	0.633	0.967	0.833	0.8	0.821		1.013
38	~ ConA + SNA I	0.95	0.8	1	0.833	1	0.896		38	~ ConA + SNA I	0.789	0.6	0.944	0.833	0.8	0.821		1.204
39	~ ConA + WFL	0.867	0.633	1	0.833	0.8	0.821		39	~ ConA + WFL	0.439	0.228	0.667	0.444	0.7	0.536		1.975
40	~ ConA + WGA	0.883	0.667	1	1	0.8	0.925		40	~ ConA + WGA	0.922	0.794	1	0.833	0.9	0.857		0.958
41	~ ConA + RPL-Fuc1	0.833	0.567	1	0.667	1	0.792		41	~ ConA + RPL-Fuc1	0.75	0.556	0.917	0.722	0.8	0.75		1.111
42	~ ConA + RPL-Sia2	0.817	0.533	1	0.667	1	0.792		42	~ ConA + RPL-Sia2	0.7	0.461	0.906	1	0.4	0.786		1.167
43	~ ConA + P sel	0.8	0.533	1	0.667	0.9	0.754		43	~ ConA + P sel	0.672	0.433	0.867	0.778	0.6	0.714		1.19
44	~ ConA + HL sel	0.8	0.517	1	0.667	0.9	0.754		44	~ ConA + HL sel	0.894	0.744	0.994	0.944	0.7	0.857		0.895
45	~ ConA + HE sel	0.817	0.533	1	0.667	1	0.792		45	~ ConA + HE sel	0.822	0.611	0.978	0.722	0.9	0.786		0.994
46	~ DBA + HPyL	0.783	0.5	1	0.667	0.9	0.754		46	~ DBA + HPyL	0.806	0.594	0.972	0.889	0.8	0.857		0.971
47	~ DBA + MAA	0.833	0.583	1	0.667	0.9	0.754		47	~ DBA + MAA	0.817	0.633	0.972	0.833	0.8	0.821		1.02
48	~ DBA + PHAE	0.783	0.533	0.967	1	0.5	0.813		48	~ DBA + PHAE	0.95	0.85	1	0.944	0.9	0.929		0.824
49	~ DBA + PHAL	0.783	0.517	0.983	0.833	0.7	0.783		49	~ DBA + PHAL	0.833	0.661	0.972	0.778	0.9	0.821		0.94
50	~ DBA + RCA I	0.8	0.533	1	1	0.7	0.888		50	~ DBA + RCA I	0.878	0.706	1	0.944	0.8	0.893		0.911
51	~ DBA + SNA I	0.95	0.816	1	1	0.8	0.925		51	~ DBA + SNA I	0.828	0.639	0.972	0.833	0.8	0.821		1.147
52	~ DBA + WFL	0.833	0.6	1	1	0.6	0.85		52	~ DBA + WFL	0.833	0.65	0.972	0.833	0.8	0.821		1
53	~ DBA + WGA	0.933	0.783	1	0.833	0.9	0.858		53	~ DBA + WGA	0.933	0.817	1	1	0.7	0.893		1
54	~ DBA + RPL-Fuc1	0.783	0.5	1	1	0.7	0.888		54	~ DBA + RPL-Fuc1	0.817	0.611	0.972	0.778	0.8	0.786		0.958
55	~ DBA + RPL-Sia2	0.817	0.567	1	1	0.8	0.925		55	~ DBA + RPL-Sia2	0.833	0.644	0.978	0.944	0.7	0.857		0.981
56	~ DBA + P sel	0.783	0.533	0.983	1	0.6	0.85		56	~ DBA + P sel	0.833	0.633	1	0.944	0.8	0.893		0.94
57	~ DBA + HL sel	0.833	0.6	1	1	0.7	0.888		57	~ DBA + HL sel	0.906	0.756	1	0.944	0.8	0.893		0.919
58	~ DBA + HE sel	0.767	0.5	0.983	0.833	0.7	0.783		58	~ DBA + HE sel	0.872	0.728	0.978	0.722	1	0.821		0.88
59	~ HPyL + MAA	0.8	0.55	0.983	1	0.5	0.813		59	~ HPyL + MAA	0.756	0.55	0.922	0.722	0.8	0.75		1.058
60	~ HPyL + PHAE	0.767	0.483	0.983	0.833	0.7	0.783		60	~ HPyL + PHAE	0.961	0.883	1	0.833	1	0.893		0.798
61	~ HPyL + PHAL	0.483	0.183	0.783	0.667	0.5	0.604		61	~ HPyL + PHAL	0.711	0.494	0.911	0.556	0.9	0.679		0.679
62	~ HPyL + RCA I	0.7	0.4	0.933	0.667	0.8	0.717		62	~ HPyL + RCA I	0.822	0.628	0.967	0.944	0.6	0.821		0.852
63	~ HPyL + SNA I	0.95	0.817	1	1	0.8	0.925		63	~ HPyL + SNA I	0.717	0.517	0.906	0.556	0.9	0.679		1.325
64	~ HPyL + WFL	0.9	0.7	1	0.833	0.9	0.858		64	~ HPyL + WFL	0.717	0.478	0.922	0.667	0.8	0.714		1.255
65	~ HPyL + WGA	0.983	0.9	1	1	0.9	0.963		65	~ HPyL + WGA	0.906	0.761	1	0.833	0.9	0.857		1.085
66	~ HPyL + RPL-Fuc1	0.5	0.217	0.8	0.5	0.7	0.575		66	~ HPyL + RPL-Fuc1	0.767	0.567	0.944	0.778	0.8	0.786		0.652
67	~ HPyL + RPL-Sia2	0.667	0.333	0.95	0.833	0.6	0.746		67	~ HPyL + RPL-Sia2	0.744	0.511	0.944	0.833	0.7	0.786		0.897
68	~ HPyL + P sel	0.617	0.317	0.867	1	0.4	0.775		68	~ HPyL + P sel	0.717	0.456	0.922	0.944	0.5	0.786		0.861
69	~ HPyL + HL sel	0.583	0.267	0.85	0.667	0.6	0.642		69	~ HPyL + HL sel	0.911	0.778	1	1	0.7	0.893		0.64
70	~ HPyL + HE sel	0.583	0.3	0.867	0.833	0.5	0.708		70	~ HPyL + HE sel	0.822	0.628	0.967	0.722	0.9	0.786		0.709
71	~ MAA + PHAE	0.85	0.617	1	0.833	0.8	0.821		71	~ MAA + PHAE	0.944	0.833	1	0.944	0.9	0.929		0.9

72	~ MAA + PHAL	0.817	0.567	1	1	0.7	0.888		72	~ MAA + PHAL	0.733	0.539	0.9	0.556	1	0.714		1.115
73	~ MAA + RCA I	0.8	0.517	0.967	1	0.6	0.85		73	~ MAA + RCA I	0.839	0.661	0.978	1	0.6	0.857		0.954
74	~ MAA + SNA I	0.95	0.8	1	0.833	1	0.896		74	~ MAA + SNA I	0.811	0.633	0.956	0.667	1	0.786		1.171
75	~ MAA + WFL	0.883	0.6	1	0.833	1	0.896		75	~ MAA + WFL	0.733	0.544	0.894	0.5	1	0.679		1.205
76	~ MAA + WGA	0.867	0.667	1	1	0.8	0.925		76	~ MAA + WGA	0.917	0.772	1	1	0.8	0.929		0.945
77	~ MAA + RPL-Fuc1	0.767	0.5	0.967	1	0.5	0.813		77	~ MAA + RPL-Fuc1	0.761	0.567	0.922	0.667	0.9	0.75		1.008
78	~ MAA + RPL-Sia2	0.783	0.5	0.967	1	0.5	0.813		78	~ MAA + RPL-Sia2	0.728	0.5	0.911	0.778	0.7	0.75		1.076
79	~ MAA + P sel	0.917	0.733	1	1	0.8	0.925		79	~ MAA + P sel	0.75	0.544	0.917	0.778	0.7	0.75		1.223
80	~ MAA + HL sel	0.75	0.467	0.967	0.833	0.6	0.746		80	~ MAA + HL sel	0.906	0.756	1	1	0.7	0.893		0.828
81	~ MAA + HE sel	0.833	0.583	0.983	1	0.6	0.85		81	~ MAA + HE sel	0.817	0.628	0.972	0.722	0.9	0.786		1.02
82	~ PHAE + PHAL	0.667	0.333	1	0.5	1	0.688		82	~ PHAE + PHAL	0.944	0.844	1	0.778	1	0.857		0.707
83	~ PHAE + RCA I	0.683	0.4	0.917	1	0.4	0.775		83	~ PHAE + RCA I	0.95	0.856	1	0.944	0.9	0.929		0.719
84	~ PHAE + SNA I	1	1	1	1	1	1		84	~ PHAE + SNA I	0.978	0.911	1	1	0.9	0.964		1.022
85	~ PHAE + WFL	0.817	0.483	1	0.833	0.9	0.858		85	~ PHAE + WFL	0.95	0.85	1	0.944	0.9	0.929		0.86
86	~ PHAE + WGA	0.883	0.667	1	0.833	0.9	0.858		86	~ PHAE + WGA	0.95	0.833	1	0.944	0.9	0.929		0.929
87	~ PHAE + RPL-Fuc1	0.767	0.467	0.983	0.667	0.8	0.717		87	~ PHAE + RPL-Fuc1	0.956	0.867	1	0.889	0.9	0.893		0.802
88	~ PHAE + RPL-Sia2	0.633	0.3	0.95	0.5	1	0.688		88	~ PHAE + RPL-Sia2	0.939	0.828	1	0.833	1	0.893		0.674
89	~ PHAE + P sel	0.667	0.333	0.95	0.667	0.8	0.717		89	~ PHAE + P sel	0.956	0.861	1	0.944	0.9	0.929		0.698
90	~ PHAE + HL sel	0.733	0.466	0.967	0.667	0.8	0.717		90	~ PHAE + HL sel	0.989	0.95	1	1	0.9	0.964		0.741
91	~ PHAE + HE sel	0.7	0.383	0.95	0.667	0.8	0.717		91	~ PHAE + HE sel	0.939	0.833	1	0.944	0.9	0.929		0.745
92	~ PHAL + RCA I	0.7	0.4	0.933	1	0.4	0.775		92	~ PHAL + RCA I	0.85	0.689	0.972	0.833	0.8	0.821		0.824
93	~ PHAL + SNA I	0.95	0.8	1	1	0.8	0.925		93	~ PHAL + SNA I	0.744	0.544	0.928	0.778	0.8	0.786		1.277
94	~ PHAL + WFL	0.917	0.733	1	0.833	0.9	0.858		94	~ PHAL + WFL	0.544	0.317	0.756	0.278	1	0.536		1.686
95	~ PHAL + WGA	0.9	0.717	1	0.833	0.9	0.858		95	~ PHAL + WGA	0.922	0.778	1	0.889	0.9	0.893		0.976
96	~ PHAL + RPL-Fuc1	0.517	0.217	0.833	0.667	0.6	0.642		96	~ PHAL + RPL-Fuc1	0.706	0.5	0.878	0.5	1	0.679		0.732
97	~ PHAL + RPL-Sia2	0.633	0.333	0.9	0.667	0.7	0.679		97	~ PHAL + RPL-Sia2	0.717	0.506	0.9	0.611	0.8	0.679		0.883
98	~ PHAL + P sel	0.617	0.317	0.884	0.833	0.6	0.746		98	~ PHAL + P sel	0.633	0.372	0.856	0.778	0.6	0.714		0.975
99	~ PHAL + HL sel	0.6	0.3	0.9	0.667	0.7	0.679		99	~ PHAL + HL sel	0.894	0.761	0.994	0.944	0.7	0.857		0.671
100	~ PHAL + HE sel	0.683	0.4	0.917	1	0.5	0.813		100	~ PHAL + HE sel	0.828	0.644	0.978	0.722	0.9	0.786		0.825
101	~ RCA I + SNA I	0.983	0.9	1	1	0.9	0.963		101	~ RCA I + SNA I	0.906	0.778	0.994	0.833	0.9	0.857		1.085
102	~ RCA I + WFL	0.75	0.433	0.967	0.5	1	0.688		102	~ RCA I + WFL	0.811	0.6	0.967	0.944	0.6	0.821		0.925
103	~ RCA I + WGA	0.883	0.667	1	1	0.8	0.925		103	~ RCA I + WGA	0.894	0.739	1	1	0.8	0.929		0.988
104	~ RCA I + RPL-Fuc1	0.733	0.45	1	0.833	0.8	0.821		104	~ RCA I + RPL-Fuc1	0.85	0.683	0.967	0.833	0.8	0.821		0.862
105	~ RCA I + RPL-Sia2	0.7	0.417	0.933	0.667	0.8	0.717		105	~ RCA I + RPL-Sia2	0.817	0.617	0.967	0.833	0.8	0.821		0.857
106	~ RCA I + P sel	0.817	0.533	1	0.833	0.8	0.821		106	~ RCA I + P sel	0.85	0.678	1	1	0.7	0.893		0.961
107	~ RCA I + HL sel	0.717	0.417	0.933	1	0.4	0.775		107	~ RCA I + HL sel	0.961	0.883	1	1	0.8	0.929		0.746

108	~ RCA I + HE sel	0.633	0.333	0.883	1	0.5	0.813		108	~ RCA I + HE sel	0.9	0.75	1	0.944	0.8	0.893		0.703
109	~ SNA I + WFL	1	1	1	1	1	1		109	~ SNA I + WFL	0.778	0.594	0.939	0.611	1	0.75		1.285
110	~ SNA I + WGA	1	1	1	1	1	1		110	~ SNA I + WGA	0.906	0.744	1	0.944	0.8	0.893		1.104
111	~ SNA I + RPL-Fuc1	0.967	0.867	1	0.833	1	0.896		111	~ SNA I + RPL-Fuc1	0.733	0.544	0.894	0.556	1	0.714		1.319
112	~ SNA I + RPL-Sia2	0.95	0.8	1	0.833	1	0.896		112	~ SNA I + RPL-Sia2	0.756	0.55	0.922	0.778	0.8	0.786		1.257
113	~ SNA I + P sel	0.95	0.8	1	1	0.8	0.925		113	~ SNA I + P sel	0.722	0.494	0.889	0.444	1	0.643		1.316
114	~ SNA I + HL sel	0.983	0.9	1	1	0.9	0.963		114	~ SNA I + HL sel	0.9	0.756	0.989	1	0.7	0.893		1.092
115	~ SNA I + HE sel	0.933	0.75	1	1	0.8	0.925		115	~ SNA I + HE sel	0.833	0.644	0.978	0.889	0.7	0.821		1.12
116	~ WFL + WGA	0.9	0.717	1	1	0.7	0.888		116	~ WFL + WGA	0.906	0.75	1	0.944	0.8	0.893		0.993
117	~ WFL + RPL-Fuc1	0.883	0.667	1	0.833	0.9	0.858		117	~ WFL + RPL-Fuc1	0.717	0.511	0.883	0.667	0.8	0.714		1.232
118	~ WFL + RPL-Sia2	0.767	0.467	1	0.667	0.9	0.754		118	~ WFL + RPL-Sia2	0.717	0.494	0.906	0.944	0.5	0.786		1.07
119	~ WFL + P sel	0.783	0.5	1	0.5	1	0.688		119	~ WFL + P sel	0.678	0.444	0.872	0.833	0.6	0.75		1.155
120	~ WFL + HL sel	0.833	0.533	1	0.833	0.9	0.858		120	~ WFL + HL sel	0.894	0.739	0.995	1	0.7	0.893		0.932
121	~ WFL + HE sel	0.75	0.467	0.967	0.5	1	0.688		121	~ WFL + HE sel	0.85	0.672	0.989	0.833	0.9	0.857		0.882
122	~ WGA + RPL-Fuc1	0.867	0.667	1	0.833	0.8	0.821		122	~ WGA + RPL-Fuc1	0.906	0.733	1	0.944	0.8	0.893		0.957
123	~ WGA + RPL-Sia2	0.85	0.6	1	1	0.6	0.85		123	~ WGA + RPL-Sia2	0.911	0.778	0.994	0.833	0.9	0.857		0.933
124	~ WGA + P sel	0.85	0.6	1	0.833	0.9	0.858		124	~ WGA + P sel	0.9	0.733	1	1	0.8	0.929		0.944
125	~ WGA + HL sel	0.883	0.683	1	0.833	0.9	0.858		125	~ WGA + HL sel	0.983	0.933	1	0.944	1	0.964		0.898
126	~ WGA + HE sel	0.9	0.7	1	0.833	0.9	0.858		126	~ WGA + HE sel	0.922	0.778	1	1	0.8	0.929		0.976
127	~ RPL-Fuc1 + RPL-Sia2	0.617	0.3	0.9	0.667	0.8	0.717		127	~ RPL-Fuc1 + RPL-Sia2	0.689	0.478	0.861	0.5	0.9	0.643		0.896
128	~ RPL-Fuc1 + P sel	0.617	0.317	0.883	0.833	0.5	0.708		128	~ RPL-Fuc1 + P sel	0.728	0.517	0.894	0.556	0.9	0.679		0.848
129	~ RPL-Fuc1 + HL sel	0.633	0.333	0.884	0.833	0.5	0.708		129	~ RPL-Fuc1 + HL sel	0.917	0.789	1	1	0.7	0.893		0.69
130	~ RPL-Fuc1 + HE sel	0.683	0.4	0.933	0.833	0.7	0.783		130	~ RPL-Fuc1 + HE sel	0.85	0.667	0.983	0.778	0.9	0.821		0.804
131	~ RPL-Sia2 + P sel	0.667	0.383	0.9	0.833	0.5	0.708		131	~ RPL-Sia2 + P sel	0.672	0.439	0.861	0.833	0.5	0.714		0.993
132	~ RPL-Sia2 + HL sel	0.617	0.283	0.883	0.833	0.5	0.708		132	~ RPL-Sia2 + HL sel	0.906	0.761	0.994	0.944	0.7	0.857		0.681
133	~ RPL-Sia2 + HE sel	0.65	0.367	0.9	0.833	0.5	0.708		133	~ RPL-Sia2 + HE sel	0.828	0.622	0.978	0.778	0.9	0.821		0.785
134	~ P sel + HL sel	0.667	0.367	0.917	1	0.4	0.775		134	~ P sel + HL sel	0.889	0.728	1	1	0.7	0.893		0.75
135	~ P sel + HE sel	0.75	0.467	0.967	1	0.6	0.85		135	~ P sel + HE sel	0.833	0.656	0.967	0.833	0.8	0.821		0.9
136	~ HL sel + HE sel	0.6	0.3	0.867	1	0.3	0.738		136	~ HL sel + HE sel	0.922	0.8	0.994	0.833	0.9	0.857		0.651
